# Proteomic analysis of exosomes derived from human lymphoma cells

**DOI:** 10.1186/s40001-014-0082-4

**Published:** 2015-01-29

**Authors:** Ye Yao, Wei Wei, Jing Sun, Linjun Chen, Xiaohui Deng, Liyuan Ma, Siguo Hao

**Affiliations:** Department of Hematology, Xinhua Hospital Affiliated to Shanghai Jiaotong University, School of Medicine, Shanghai, China

**Keywords:** Exosomes, Lymphoma cells, Shotgun, Gene ontology, Kyoto encyclopedia of genes and genomes, Mass spectrometry

## Abstract

**Background:**

Exosomes secreted by tumor cells contain specific antigens that may have immunotherapeutic purposes. The aim of this study was to characterize the proteomic content of lymphoma cell-derived exosomes (LCEXs).

**Methods:**

In this study, exosomes derived from Raji cells (EXO_Raji_) were purified and proteins of EXO_Raji_ were separated by one-dimensional sodium dodecyl sulfate polyacrylamide gel electrophoresis. Protein bands were identified by mass spectrometry. The protein components of EXO_Raji_ were analyzed using shotgun technology, and the function proteins of EXO_Raji_ were defined and described using the Gene Ontology (GO) database and Kyoto Encyclopedia of Genes and Genomes (KEGG) analysis.

**Results:**

A total of 197 proteins were identified in EXO_Raji_; 139 proteins were also identified in Raji cells, showing an overlap of 70.56% of the total proteins in EXO_Raji_. Interestingly, the remaining 58 proteins were unique to EXO_Raji_. The GO database and KEGG were used to define and describe the function of proteins. The data showed that some important proteins involved in antigen procession and presentation as well as cell migration and adhesion were also identified in EXO_Raji_, such as MHC-I and II, HSC70, HSP90, and ICMA-1.

**Conclusions:**

LCEXs express a discrete set of proteins involved in antigen presentation and cell migration and adhesion, suggesting that LCEXs play an important role in the regulation of immunity and interaction between lymphoma cells and their microenvironment. LCEXs harbor most of the proteins of lymphoma cells and could be one of the sources of lymphoma-associated antigens for immunotherapeutic purposes.

## Background

Tumor cells can release exosomes. Exosomes are a type of vesicle secreted by late endosomes in eukaryotic cells and loaded with cell membrane molecules, microRNAs, and proteins [1-4]. Studies on the protein composition of exosomes of dendritic cells (DCs) showed that loaded proteins are mainly involved in regulation of cell physiological activities [5-7]. Moreover, molecules involved in antigen presentation such as major histocompatibility complex (MHC)-I and MHC-II have also been detectable in DC-derived exosomes (DEXs) [6,7].

Although DEXs can induce antigen-specific antitumor immunity, the biological characteristics of tumor cell-derived exosomes (TEXs), especially those from lymphoma cells, remain unclear. Our previous studies and others revealed that DEXs and TEXs can be taken up by DCs and transfer tumor antigens to DCs *in vitro* and that TEX-targeted DCs can induce stronger antigen-specific antitumor immunity than TEXs alone [5,8-10]. These studies collectively suggest that TEXs harbor tumor antigens and are potential targets in the development of effective antitumor vaccines [11,12].

Burkitt lymphoma (BL) is a highly aggressive B-cell lymphoma with an extremely short cell doubling time and usually presents in extranodal sites or as an acute leukemia [13]. Here, BL tumor cells were chosen as a model system to study the protein components of exosomes derived from human lymphoma cells because few data are available on the protein components of exosomes derived from this tumor type.

Proteomic technologies such as two-dimensional electrophoresis and mass spectrometry (MS) enable us to comprehensively study the protein composition of TEXs. Our previous studies demonstrated that TEXs harbor tumor cell-associated antigens and can induce tumor antigen-specific antitumor immunity [5,10].

Similar to other tumor cells, lymphoma cells can release exosomes [10]. However, the protein constituents of LCEXs have still not been identified. In this study, proteins isolated from EXO_Raji_ were separated by one-dimensional SDS-PAGE, and protein bands were identified by mass spectrometry. The protein components of EXO_Raji_ were analyzed using shotgun technology. The GO database and KEGG analysis were used to define and describe the function of proteins.

## Methods

### Chemicals and reagents

AIM-V serum-free conditioning medium, RPMI 1640 medium, and fetal bovine serum were purchased from Invitrogen (Grand Island, Shanghai, China). Deuterium oxide (D_2_O) was purchased from Tenglong Weibo Technology (Qingdao, China). Sodium dodecyl sulfate polyacrylamide gel electrophoresis (SDS-PAGE) buffer and 3-[(3-cholamidopropyl) dimethylamino]-1- propanesulfonate (CHAPS) were purchased from Sigma Aldrich (Sigma Aldrich, Shanghai, China). TPCK-trypsin was purchased from Promega (Promega, Shanghai, China). Acetonitrile (GC purity) was purchased from Merck (Merck, Shanghai, China). Lysis buffer, 4× separating gel buffer, and balanced salt solution were sterilized by passing through a 0.22-μm membrane filter before sub-packaging and storing at −80°C.

### Preparation of exosome purification

Raji cells were cultured in RPMI 1640 medium containing 10% inactivated fetal bovine serum at 37°C in a 5% CO_2_ humidified incubator. After 24–72 h in serum-free conditioned medium, cell viability was >95% as determined by a trypan blue exclusion assay. Exosomes were prepared as previously described [10,14]. The supernatant was centrifuged at a series of successively lower forces to remove cells and cellular debris. The resultant clarified supernatant was concentrated by centrifugation at 100,000 *g* at 4°C for 60 min. The pellets were washed with 0.01 M phosphate-buffered saline, transferred to a 10-ml ultracentrifuge tube underlaid with a 1.5-ml 30% sucrose/D_2_O density cushion (density: 1.210 g/cm^3^), and ultracentrifuged (Beckman Coulter, Shanghai, China) at 100,000 *g* at 4°C for 1 h. Finally, the exosomes were concentrated by centrifugation at 1,000 *g* for 60 min in a pre-rinsed 100-kDa molecular weight Ultra capsule filter (Millipore, Billerica, Shanghai, China). The concentration of exosomal proteins was determined using a Quick Start™ Bradford Protein Assay Kit (Bio-Rad®, Shanghai, China). The exosomes were stored at −80°C until use.

### Morphological characteristics of EXO_Raji_

Exosomes derived from Raji cells (10 μg) were washed in cacodylate buffer, fixed in 2.5% glutaraldehyde (Polysciences, Shanghai, China) in cacodylate buffer overnight at 4°C, dehydrated by graded alcohol processing, and flat embedded in LX-112 epoxy resin. Sections were cut with an ultramicrotome. Mounted sections were collected on copper grids, stained with a saturated solution of uranyl acetate, and submitted for observation and imaging under a Philips CM12 transmission electron microscope (TEM) (Philips, Shanghai, China) [15]. To characterize the exosomes, ER-residing molecules such as ER-residing protein Grp94 in exosomes derived from Raji cells were examined by Western blot.

### Two-dimensional SDS-PAGE

Isoelectronic focusing (IEF) was performed with 18-cm immobilized strips with pH values from 3–10 and 4–9 in an IPGphor electrophoresis device (Amersham Bioscience, Shanghai, China). Briefly, samples of EXO_Raji_ and Raji cells were diluted into 350 ml buffer and loaded onto an IPG strip. IEF was carried out at 20°C according to the following schedule: 12 h at 30 V, 2 h at 100 V, 1 h at 500 V, 1 h at 1,000 V, 1 h of a linear gradient to 8,000 V, and 10 h at 8,000 V. After IEF, the strips were equilibrated in 6 M urea, 30% glycerol, 2% SDS, and 50 mM Tris-HCl (pH 6.8) containing 1% 1,4-dithiothreitol for 15 min followed by 2.5% (w/v) iodoacetamide. The strips were transferred onto the gel and sealed in place with 1% agarose. The two-dimensional SDS-PAGE was performed using 12% polyacrylamide gels at 20 mA and 16 V on Bio-Rad Protein II (Bio-Rad, Shanghai, China) and was terminated when the bromophenol blue front had migrated to the lower end of the gels. The gels were then stained with silver [16] and scanned by the ImageScanner (Amersham Pharmacia, Shanghai, China).

### One-dimensional protein electrophoresis

One-dimensional electrophoresis of EXO_Raji_ on 10% SDS-PAGE gels was performed according to the manufacturer’s instructions (BioRad Laboratories, Shanghai, China). Then 10 μg of each sample was taken up in 8 M urea, 2% CHAPS, 20 mM dithiothreitol, and 0.01% bromophenol blue and transferred onto 1.0-mm-thick 10% SDS-PAGE gels. A constant current of 7 mA per gel was applied at 10°C. After 16 h, the gels were stained using the Novex Colloidal Blue Staining Kit according to the manufacturer’s instructions (Invitrogen, Shanghai, China).

### Enzymatic digestion of protein and capillary

Stained electrophoresis gels loaded with Raji cells and EXO_Raji_ were excised into four parts and submitted for enzymatic digestion. Proteins were first enzymolyzed into peptides. Enzymatic digestion of proteins in the gels was performed as previously described [14]. Briefly, the Ettan™ MDLC system (GE Healthcare, Shanghai, China) was used to desalt and separate the tryptic peptides mixtures. In this system, samples are desalted on reversed phase (RP) trap columns (Agilent Technologies, Shanghai, China) and subsequently separated on RP columns (Column Technology Inc., Shanghai, China). Mobile phase A (0.1% formic acid in HPLC-grade water) and mobile phase B (0.1% formic acid in acetonitrile) were selected. The tryptic peptide mixture (20 μg) was loaded onto the column, and separation was performed at a flow rate of 2 μl/min with a linear gradient of 4–50% B for 120 min. A Finnigan LTQ™ linear ion trap MS (Thermo Electron, Shanghai, China) equipped with an electrospray interface was connected to the LC setup to detect eluted peptides. Data-dependent MS/MS spectra were obtained simultaneously. Each scan cycle consisted of one full MS scan in the profile mode followed by five MS/MS scans in the centroid mode with the following Dynamic Exclusion™ settings: repeat count: 2; repeat duration: 30 s; exclusion duration: 90 s. Each sample was analyzed in triplicate.

### Mass spectrometry data acquisition and analysis

MS/MS spectra were automatically searched against the non-redundant International Protein Index human protein database (version 3.26, 67 687 entries) using the Bioworks Browser 3.1. The peptides were constrained to be tryptic, and up to two missed cleavages were allowed. Carbamidomethylation of cysteines was treated as a fixed modification, whereas methionine residue oxidation was considered a variable modification. The mass tolerances allowed for precursor and fragment ions were 2.0 and 0.0 Da, respectively. The protein identification criteria were based on Delta CN (≥0.1) and cross-correlation scores (X_corr_, one charge ≥1.9; two charges ≥2.2; three charges ≥3.75).

### Western blotting

Western blotting was performed following one-dimensional SDS-PAGE as follows. Protein samples from EXO_Raji_ and Raji cells were electroblotted onto Immobilon P membranes (Millipore Corp, Shanghai, China) and incubated with specific antibodies, followed by horseradish peroxidase-conjugated secondary antibodies, and detected using SuperSignal West Pico chemiluminescent substrate (Pierce Perbio Science, Shanghai, China). Antibodies used in this study to confirm the proteins detected by MS were: anti-HSC70 (clone 13D3; Thorma Scientific, Shanghai, China), anti-ICAM-1 (clone 9H21L19; Thorma Scientific, Shanghai, China), and anti-actin (clone 15G5A11/E2, Thorma Scientific, Shanghai, China).

## Results

### Morphology of exosomes derived from lymphoma cells

**Figure 1:**
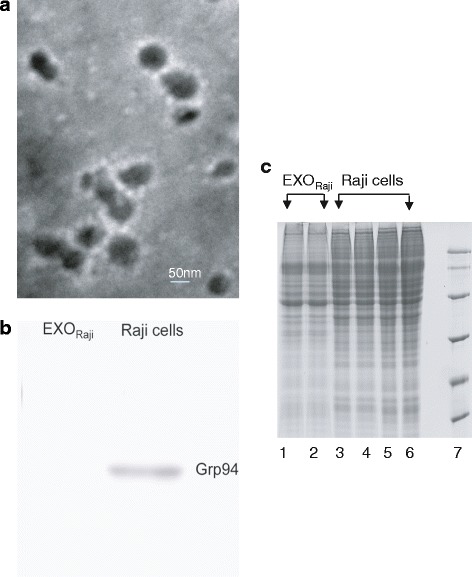
**Morphology of EXO**_**Raji**_ **and one-dimensional electrophoresis of samples from EXO**_**Raji**_ **and Raji cells. a**. Transmission electron microscope image of EXO_Raji_ (×100 K). **b**. Western blot analysis demonstrating the absence of Grp94 in EXO_Raji_. **c**. Protein samples from EXO_Raji_ and Raji cells were separated by SDS-PAGE for further MS analysis. *Lanes 1 and 2* represent Raji cell-derived exosomes, and *lanes 3 to 6* represent Raji cells.

We first assessed whether exosomes derived from Raji cells were similar to those released by other tumor cell lines. The supernatant was centrifuged at up to 100,000 *g*, and the pellet was similar to previously reported exosomes, as determined by electron microscopy [17]. The vesicles were <100 nm in diameter and had a dimpled, cup-shaped morphology, characteristic of exosomes (see Figure 1a). To characterize EXO_Raji_, we also examined the presence of the ER-residing protein Grp94 by Western blot. Data showed that Grp94 was absent in EXO_Raji_ (see Figure 1b). Taken together, these results indicate that vesicles obtained from cell-free supernatants of Raji lymphoma cells exhibit characteristic biophysical properties of exosomes.

### Mass spectrometric analysis of proteins in EXO_Raji_

**Figure 2:**
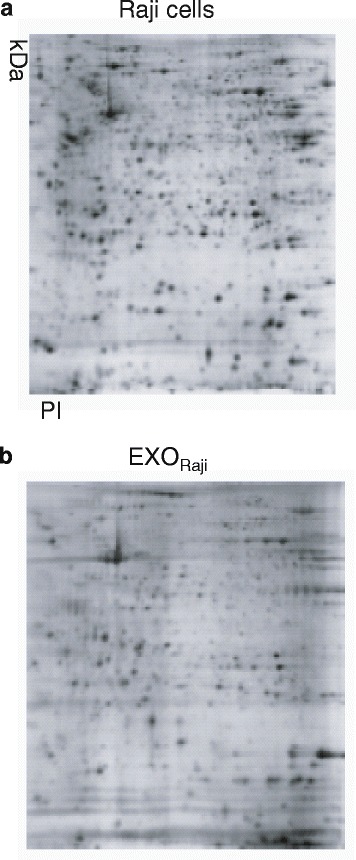
**Two-dimensional electrophoresis of samples from Raji cells (a) and EXO**_**Raji**_ **(b).**

Protein samples were then separated by one-dimensional SDS-PAGE for further MS analysis. The gels were stained with Coomassie Blue R-250, and protein bands were digested for capillary high-performance liquid chromatography analysis. As shown in Figure 1, protein bands showed similar patterns between the preparations from EXO_Raji_ and Raji cells. Two-dimensional electrophoresis was also used to compare the protein contents from EXO_Raji_ and Raji cells (see Figure 2). Although the protein spots of EXO_Raji_ were slightly less than those of the Raji cells, more than 70% of the protein spots in Raji cells were also found in EXO_Raji_. To further explore the proteomic characteristics of EXO_Raji_, EXO_Raji_ samples were separated by one-dimensional SDS-PAGE for further MS analysis, the base peak maps of Raji cells and EXO_Raji_ were collected according to the mass-to-charge ratios of the peptides and peptide fragments.

**Figure 3:**
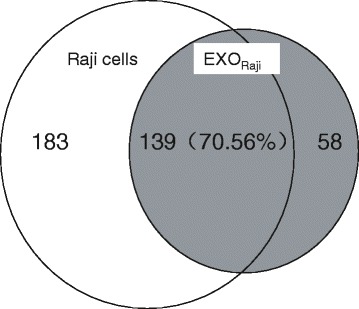
**Distribution of identified proteins in Raji cells and EXO**_**Raji**_ **.** Shotgun protein identification reveals 322 proteins in Raji cell samples (*white*) and 197 in EXO_Raji_ samples (*gray*); 139 proteins are identified in both samples, accounting for 70.56% of the total identified proteins in EXO_Raji_ samples, while the remaining 58 proteins are unique to EXO_Raji_.

All collected base peak data were analyzed by DeCyder MS and BuildSummary 4.9.0 software to output all identified proteins. According to shotgun protein identification, 322 proteins were identified in Raji cell samples, while 197 proteins were identified in EXO_Raji_. Among the proteins in EXO_Raji_ samples, 139 were identified in both samples, showing an overlap of 70.56% of the total proteins in EXO_Raji_ samples, indicating that more than 70% of proteins in EXO_Raji_ were derived from their parental cells. Interestingly, the remaining 58 proteins, accounting for about 30% of proteins in EXO_Raji_, were not identified in Raji cells, indicating that some proteins in EXO_Raji_ may be obtained from the extracellular microenvironment or enriched in the process of exosome development (see Figure 3). Most of these proteins are a composition of HLA class I and II molecules, such as HLA-B, HLA-C histocompatibility antigen, B-15 alpha chain, B-39 alpha, A-26 alpha China, HLA-DQA1 MHC class II antigen, HLA class II histocompatibility antigen, DQ(1) beta China, HLA-C antigen, Cw-4 alpha and Cw-3 alpha chain, HLA-DPB1 major histocompatibility complex, class II, DP beta1, CD81, CD82 antigen and intercellular adhesion molecule 1, etc. These proteins play an important role in antigen processing and presentation and regulation of cell interactions.

### Analysis of protein function in EXO_Raji_ using GO analysis

Next, we analyzed the function of proteins in EXO_Raji_ according to the biological process, molecular function, and cell components with GO analysis. As shown in Table 1, among the 139 proteins in EXO_Raji_, 46.7% and 43.2% were involved in localization and establishment of localization, respectively; 41.7% of the proteins were involved in response to stimulus, 38.1% of the proteins were involved in cellular component organization, and more than 20% of proteins were involved in immune system processes. Similarly, among 58 proteins unique to EXO_Raji_, more than 30% were involved in localization, establishment of localization, and response to stimulus (see Table 2), indicating that LCEX plays an important role in biological process including response to stimuli and establishment of localization. In terms of molecular function, all proteins identified in EXO_Raji_ were involved in protein binding, more than 45% of which were involved in nucleotide binding, and 20% were involved in nucleoside binding and hydrolase activity. Similarly, more than 60% of the proteins unique to EXO_Raji_ were involved in protein binding (Table 2), suggesting that LCEX plays an important role in protein binding.

**Table 1:** **Categories of proteins identified in Raji cells and EXO**_**Raji**_ **by GO analysis**

**Biological process**				**Molecular function**			
	**E&R**	**EXO**_**Raji**_	**Raji cells**		**E&R**	**EXO**_**Raji**_	**Raji cells**
Response to stimulus	45	60	108	Hydrolase activity	39	43	82
Immune system process	18	32	16	Nucleoside binding	40	42	91
Cell killing	3	3	3	Nucleotide binding	58	60	143
Localization	46	65	77	Protein binding	105	139	250
Multi-organism process	11	20	25	Nitric-oxide synthase regulator activity	2	2	2
Establishment of localization	43	62	74	Proteasome activator activity	2	2	2
Cellular component organization	44	57	86	Nucleobase binding	2	2	3
Cellular component biogenesis	23	27	58	Cell surface binding	4	5	4
**Cell component**				Carboxylic acid binding	5	5	7
	**E&R**	**EXO**_**Raji**_	**Raji cells**	Translation factor activity	5	5	10
Ribonucleo protein complex	8	10	61	Lyase activity	8	8	11
Protein complex	47	52	98	Cofactor binding	9	9	15
Organelle lumen	24	24	100				
Non-membrane bounded	45	51	113				
Organelle part	52	56	162				
Intracellular organelle	86	105	241				
Intracellular part	107	139	292				
Organelle membrane	14	17	33				
Vesicle	24	32	34				
Organelle envelope	11	14	34				

The protein constituents of Raji cell and EXO_Raji_ were analyzed by GO software according to the biological pathways, molecular function, and cell components. Table 1 shows the analysis results. The numbers of protein kinds presented in each sample or in both samples were listed.

**Table 2:** **Category of proteins uniquely identified in EXO**_**Raji**_ **by GO analysis**

**Molecular function**		**Biological process**		**Cell component**	
Pattern binding	3	Reproduction	9	Protein-lipid complex	3
Carbohydrate binding	6	Reproductive process	9	Protein complex	13
Signal transducer activity	14	Immune system process	11	Cell surface	5
Protein binding	36	Establishment of localization	18	Extracellular space	7
		Localization	20	Vesicle	8
		Response to stimulus	22		
		Multiorganism process	11		

The protein constituents of 58 kinds of proteins unique to EXO_Raji_ were analyzed by GO software according to the biological pathways, molecular function, and cell components. Table 2 shows the analysis results. The numbers of proteins presented according to the categories of functions were listed.

Other and our previous studies have demonstrated that exosomes may harbor proteins and associated antigens of their parental cells [5,10,18,19]. In this study, more than 90% of the proteins in EXO_Raji_ were involved in intracellular components, approximately 75% of proteins were involved in intracellular organelles, and approximately 47% of proteins were involved in intracellular organelle components and organelle components. Among the proteins uniquely identified in EXO_Raji_, more than 40% were involved in the membrane and membrane components. Taken together, these data suggest that LCEX is involved in various biological activities associated with biological processes, molecular functions, and cellular components, which provides further evidence that LCEXs harbor intracellular and membrane proteins from their parental cells.

### KEGG pathway analysis

**Figure 4:**
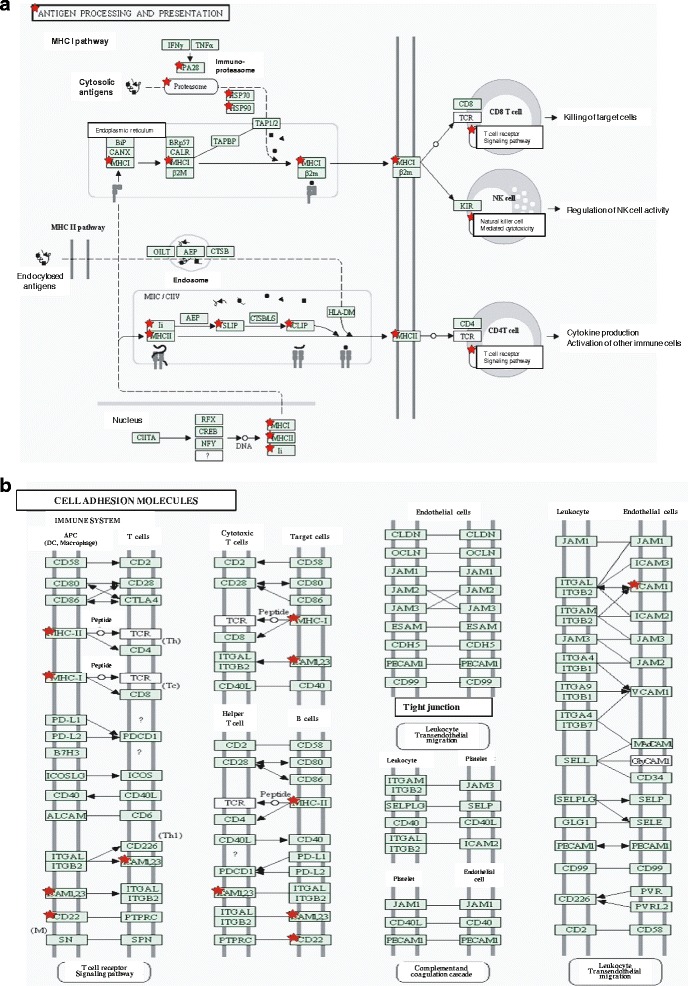
**KEGG analysis in EXO**_**Raji**_ **reveals proteins involved in antigen processing and presenting (a) and cell adhesion (b).** KEGG used for pathway analysis of proteins expressed in EXO_Raji_ shows 12 proteins involved in antigen processing and presenting and eight proteins involved in cell adhesion. Indicated molecules are marked with stars.

**Figure 5:**
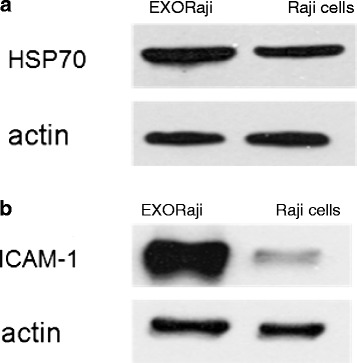
**The presence of actin, HSP70 (a), and ICAM-1 (b) is confirmed by Western blot analysis of EXO**_**Raji**_ **.** The primary antibodies were anti-actin (1:10,000) and anti-HSP70 (1:1,000), followed by horseradish peroxidase-conjugated goat anti-mouse IgG1. Blots were incubated with SuperSignal West Pico chemiluminescent substrate and exposed to Hyperfilm ECL (Amersham Biosciences, Shanghai, China).

The KEGG provides an operating platform based on the building blocks of genome information (i.e., GENES) and chemical substance information (i.e., LIGAND), linking the genome to biological systems via metabolic networks (i.e., PATHWAY), subsequently categorizing proteins according to the functional level (i.e., BRITE) [20]. In this study, KEGG pathway analysis was used to analyze the proteins identified in EXO_Raji_. We found that 12 proteins identified in EXO_Raji_ are involved in antigen processing and presenting, such as PA28, heat shock protein (HSP) 70 and 90, MHC-I, T-cell receptor, and killer-cell immunoglobulin-like receptor (see Figure 4a). Ten proteins in EXO_Raji_ are involved in several metabolic pathways such as glycolysis/gluconeogenesis, and eight proteins are involved in cell adhesion (see Figure 4b). Western blotting also confirmed the presence of HSP 70 and ICAM-1 in EXO_Raji_ (see Figure 5). Taken together, our data indicate that LCEXs harbor some immunological and adhesion molecules and may play an important role in immune regulation and cell adhesion.

## Discussion

To date, tumor exosomes have not yet been characterized. While much is known about dendritic cell-derived exosomes, no studies have reported extensive protein characterization of LCEXs. In this study, exosome-like vesicles were isolated from the culture supernatant of the Raji lymphoma cell line through successive centrifugation. Electron microscopy showed vesicles measuring 60–150 nm in diameter. Western blot revealed the absence of ER-residing protein Grp94 in EXO_Raji_, while acetyl cholinesterase activity was detectable in EXO_Raji_. Taken together, these data indicate that vesicles obtained from cell-free supernatants of Raji lymphoma cells exhibit similar biophysical properties to those of exosomes.

Mass spectrometry is a classical method in proteomics that facilitates the identification of complex protein profiles. This technique requires small samples but outputs data of high resolution with high detection sensitivity [21]. In this study, MS was used to analyze the protein components of EXO_Raji_ and to determine the differences and similarities of protein components between Raji cells and EXO_Raji_ to investigate the functional role of EXO_Raji_ proteins. More than 70% of proteins in EXO_Raji_ were derived from its parental Raji cells, consistent with the data from two-dimensional electrophoresis. These data indicate that LCEXs harbor the majority of proteins derived from the cellular components of their parental cells. Meanwhile, we also demonstrated that some of surface molecules of Raji cells, such as CD19, CD20, and CD22, also were identified in EXO_Raji_. These surface molecules of Raji cells are not only surface antigens, but also are targets of treatment; for example, rituximab, an anti-CD20 antibody, has been widely used in the treatment of adult Burkitt and Burkitt-type lymphoma or acute lymphoblastic leukemia [22]. Previous and our studies have shown that exosomes derived from mouse tumors contain a large proportion of tumor antigens and MHC class I molecules loaded with tumor peptides [9,10,23]; these studies have demonstrated that tumor cell-derived exosomes could induce tumor antigen-specific antitumor immunity. Taken together, we speculate that LCEXs may be a potential source of lymphoma cell-associated antigens for immunotherapy. However, 58 proteins were uniquely identified in EXO_Raji_, indicating that some proteins may be enriched in LCEXs and acquired from the extracellular microenvironment. LCEXs may play a role in antigen processing and presentation and regulation of cell interactions since most of the proteins in EXO_Raji_ are composed of HLA class I and II molecules and CD81, CD82, and intercellular adhesion molecule-1.

Few studies have described the functions of proteins in LCEXs. To the best of our knowledge, this is the first study to demonstrate that EXO_Raji_-derived proteins are involved in many biological activities such as response to stimuli, establishment of localization, and protein and nucleotide binding. Because the LCEX vesicle is mainly formed in the cell lumen, it contains the largest proportion of protein molecules involved in the formation of intracellular components including organelle components such as the organelle lumen. In addition, such structures contain the majority of proteins involved in the formation of intracellular components.

The KEGG provides an operating platform based on the building blocks of genome information and chemical substance information, subsequently categorizing proteins according to the functional level. In this study, the KEGG database was used to identify the detected proteins, revealing that eight of the proteins found in LCEX were cell adhesion molecules (CAMs), mainly ICAM and MHC molecules, while 12 proteins were involved in antigen processing and presenting, belonging to the heat shock cognate protein (HSP)70 and HSP90 family and PA28. These molecules had higher credibility in the identification of proteins loaded on LCEX; ICAM-1 and HSP70 indeed presented in LCEX according to our Western blotting results. Consistent with our findings, Thery *et al.* suggested that exosomes contain HSP70, while Nylandsted *et al.* reported the expression of HSP70 in endosome-lysosomal compartments [7,24]. HSPs are a group of common proteins that play a role in the cell response to elevated temperature, infection, cytokine stimulation, and other environmental stresses. HSPs are normally present in small amounts within the cytoplasm of all cells in all life forms, but can also be released into the extracellular environment in the absence of cellular necrosis [25]. The precise mechanisms by which HSPs are actively released by viable cells have not yet been elucidated, but we propose that exosomes play a role in releasing HSPs from cells. Inside cells, HSPs are involved in protein trafficking, whereby they regulate the proper folding of other proteins, maintain their correct and functional shape, and transport them from one location to another [26]. These proteins thus act as chaperones, bringing along with them small fragments or peptides derived from other proteins expressed in the cell, providing a “fingerprint” of the cell’s content [27]. Therefore, exosomes carrying large amounts of HSPs from tumor cells may be optimal candidates for cancer immunotherapy without the need to identify the antigens themselves. *In vivo,* HSPs in exosomes can be taken up by DCs and macrophages via CD91 receptor-mediated endocytosis [28,29] and then processed for presentation to the immune system in lymph nodes. Tumor-specific antigens are released from the HSP inside the cell and presented to cytotoxic T cells (CTL), or “killer cells”, which are then activated. Many studies have shown that immune cells stimulated with HSPs can eliminate different kinds of cancer cells [30-33].

While intercellular adhesion molecule-1 (ICAM-1) is a transmembrane protein, two types of extracellular ICAM-1 have been detected in cell culture supernatants as well as in the serum: a soluble form of ICAM-1 (sICAM-1) and a membranous form of ICAM-1 (mICAM-1) associated with exosomes. Previous observations have demonstrated that sICAM-1 cannot exert potent immune modulatory activity because of its low affinity for leukocyte function-associated antigen-1 (LFA-1) or membrane attack complex-1. A previous study has demonstrated that mICAM-1 on exosomes retained its topology similarly to that of cell surface ICAM-1 and could bind to leukocytes, indicating that mICAM-1 on exosomes exhibits potent immunomodulatory activity [34]. Other studies and our previous one also demonstrated that exosomes loaded with tumor antigen can be taken up by dendritic cells (DCs) and transfer tumor antigen to DCs *in vitro*; activated T cells also can recruit dendritic cell-derived exosomes via LFA-1. Taken together, LFA-1/ICAM-1 interactions play an important role in exosome uptake by antigen-presenting cells and T cells [5,35].

PA28, an 180,000-Da protein, is a proteasome activator that is strongly induced by the major immunomodulatory cytokine IFN-γ [36,37] and has been implicated in the regulation of MHC class I Ag processing [16]. PA28 may accelerate the production of MHC class I ligand from longer precursor peptides by the 20S proteasome *in vitro* [38], regulate the proteasome’s hydrolysis of small nonubiquitinated peptides as a positive allosteric effector, and modulate the proteasome-catalyzed production of antigenic peptides presented to the immune system on MHC class I molecules [16,39]. Therefore, we hypothesized that HSC70 and CAMs presented by LCEX may promote the combination, endocytosis, and internalization of LCEX into APCs in a receptor-mediated manner, thereby enhancing immune responses.

## Conclusion

In conclusion, LCEXs express a discrete set of proteins involved in antigen presentation, signal transduction, and adhesion and thereby may provide an important pathway in the communication between cells. On the other hand, LCEXs harbor most of proteins expressed in their parental lymphoma cells and may be a potential source of lymphoma cell antigens for immunotherapy.

## Abbreviations

DCs: dendritic cells
DEX: DC-derived exosome
TEXs: tumor cell-derived exosomes
MS: mass spectrometry
LCEX: lymphoma cell-derived exosomes
SDS-PAGE: sodium dodecyl sulfate polyacrylamide gel electrophoresis
HSP: heat shock protein
ICAM-1: intercellular cell adhesion molecule-1
GO: gene ontology
KEGG: Kyoto Encyclopedia of Genes and Genomes
LFA-1: leukocyte function-associated antigen-1
